# Persistent Growth of a Human Plasma-Derived Hepatitis C Virus Genotype 1b Isolate in Cell Culture

**DOI:** 10.1371/journal.ppat.1000910

**Published:** 2010-05-20

**Authors:** Erica Silberstein, Kathleen Mihalik, Laura Ulitzky, Ewan P. Plant, Montserrat Puig, Sara Gagneten, Mei-ying W. Yu, Neerja Kaushik-Basu, Stephen M. Feinstone, Deborah R. Taylor

**Affiliations:** 1 Division of Emerging and Transfusion-transmitted Diseases, Center for Biologics Evaluation and Research, Food and Drug Administration, Bethesda, Maryland, United States of America; 2 Division of Viral Products, Center for Biologics Evaluation and Research, Food and Drug Administration, Bethesda, Maryland, United States of America; 3 Division of Therapeutic Proteins, Center for Drug Evaluation and Research, Food and Drug Administration, Bethesda, Maryland, United States of America; 4 Division of Hematology, Center for Biologics Evaluation and Research, Food and Drug Administration, Bethesda, Maryland, United States of America; 5 Department of Biochemistry and Molecular Biology, University of Medicine and Dentistry of New Jersey, Newark, New Jersey, United States of America; University of Washington, United States of America

## Abstract

HCV (hepatitis C virus) research, including therapeutics and vaccine development, has been hampered by the lack of suitable tissue culture models. Development of cell culture systems for the growth of the most drug-resistant HCV genotype (1b) as well as natural isolates has remained a challenge. Transfection of cultured cells with adenovirus-associated RNA_I_ (VA RNA_I_), a known interferon (IFN) antagonist and inhibitor of dsRNA-mediated antiviral pathways, enhanced the growth of plasma-derived HCV genotype 1b. Furthermore, persistent viral growth was achieved after passaging through IFN-α/β-deficient VeroE6 cells for 2 years. Persistently infected cells were maintained in culture for an additional 4 years, and the virus rescued from these cells induced strong cytopathic effect (CPE). Using a CPE-based assay, we measured inhibition of viral production by anti-HCV specific inhibitors, including 2′-C-Methyl-D-Adenosine, demonstrating its utility for the evaluation of HCV antivirals. This virus constitutes a novel tool for the study of one of the most relevant strains of HCV, genotype 1b, which will now be available for HCV life cycle research and useful for the development of new therapeutics.

## Author Summary

Hepatitis C virus (HCV) causes a persistent infection that can lead to hepatocellular carcinoma and liver cirrhosis. Interferon (IFN)-based treatments are ineffective for some HCV genotypes. HCV research has been hampered by the lack of suitable cell culture systems. With the discovery of a unique HCV genotype 2a isolate that can replicate in the human liver cell line Huh7, some obstacles were overcome. However, there remains the need of systems to grow IFN-resistant genotypes and serum-derived isolates. Here we show that the presence of adenovirus-associated RNA_I_ (VA RNA_I_), a known IFN antagonist, permitted establishment of a persistent infection of genotype 1b in VeroE6 cells that were passaged weekly for more than 2 years. The persistent virus induces strong cytopathic effect (CPE), a feature that allowed the development of a CPE-based assay to test HCV-specific inhibitors, neutralization by anti-HCV immunoglobulins and by anti-CD81 antibody, and HCV-specific siRNA. Our system provides the first persistent culture of genotype 1b virus and a convenient assay that can be used for therapeutics development.

## Introduction

Hepatitis C virus (HCV), a member of the Flaviviridae family, is an enveloped, positive-sense RNA virus that infects approximately 170 million people worldwide. Chronic HCV infection can lead to serious liver disease, including cirrhosis and hepatocellular carcinoma. Current therapy with pegylated interferon (IFN) and ribavirin is expensive, associated with serious side effects and only effective in about 50% of treated patients. Of the six major genotypes of HCV, the relatively IFN-resistant genotypes 1a and 1b predominate in the United States, Japan and Western Europe [1].

Recent developments have advanced the HCV research field whereby a single virus isolate (cloned from a patient with a rare case of fulminant hepatitis C), JFH-1, or derivatives of that isolate have been shown to robustly replicate in the human hepatoma cell line, Huh7 [2], [3]. Full-length replicons constructed by adding the structural coding regions from another genotype 2a virus, J6 [2], were shown to not only replicate in culture, but to efficiently produce infectious viral particles [2]–[6]. Replication of the J6/JFH-1 virus in Huh7 cells was more robust in a derivative cell line, termed Huh7.5, which was selected from replicon-containing Huh7 cells after curative treatment with IFN [6], [7]. An infectious system based on the use of a Vero cell line and the pHCV-WHU-1 consensus clone (genotype 1b) was reported to produce high levels of HCV genome (>10^8^ copies/ml) with the aid of T7 polymerase provided by recombinant vaccinia virus vTF7-3 [8].

While the current cell culture systems utilize viruses that were initially replicon-derived from the JFH-1 isolate [2]–[4], [9]–[15], from HCV genotype 1b consensus clones [8], [16] or from the HCV genotype 1a prototype virus (H77-S) [10], there remains the need for a system that would be permissive for a wide variety of HCV strains found in nature. Human hepatocytes (including fetal hepatocytes) have been reported to support virus replication after RNA transfection or infection with patient sera [17], [18]. However, the use of primary cells has several technical limitations because they proliferate poorly *in vitro* and divide only a few times. Primary cultures could be maintained for longer periods of time only if the cells were immortalized by introducing oncogenes, a procedure that typically results in changes of the hepatocyte characteristics and function [17].

One approach to overcoming the obstacle of limited HCV growth in culture is to identify the mechanism of restriction. Activation of alpha/beta interferon (IFN-α/β) production is a key step in the innate response to viral infection and to the presence of double-stranded RNA (dsRNA) synthesized during replication of many viruses [19]. Several cellular dsRNA-binding proteins have been implicated in the IFN-response to infection. For instance, we have previously identified the adenosine deaminase that acts on dsRNA (ADAR1) as an IFN-α/β-induced protein that is a potent inhibitor of HCV replicon growth in cell culture [20]. ADAR1 converts adenosines in viral RNA to inosine [21], rendering the RNA inactive [20]. Both ADAR1 and the IFN-induced dsRNA-activated protein kinase (PKR) are inhibited by the small adenovirus-associated RNA (VA RNA_I_) [20], [22], [23]. When VA RNA_I_ is transfected into replicon containing Huh7 cells, it increases replication by 40-fold [20], suggesting that these IFN-induced proteins impose critical limitations to HCV replication.

In this study, we achieved growth of an HCV genotype 1b isolate by inoculating IFN-deficient cells with human plasma from an infected patient. Viral replication was stimulated further with the addition of VA RNA_I_, and led to the creation of a cell line persistently infected with HCV. More interestingly, the virus isolated from these cultures has the potential to induce cytopathic effects in the persistently infected VeroE6 cells and cause massive cell death in Huh7.5 cells.

## Results

### Construction of a persistently infected cell line, LB-piVe

**Figure 1:**
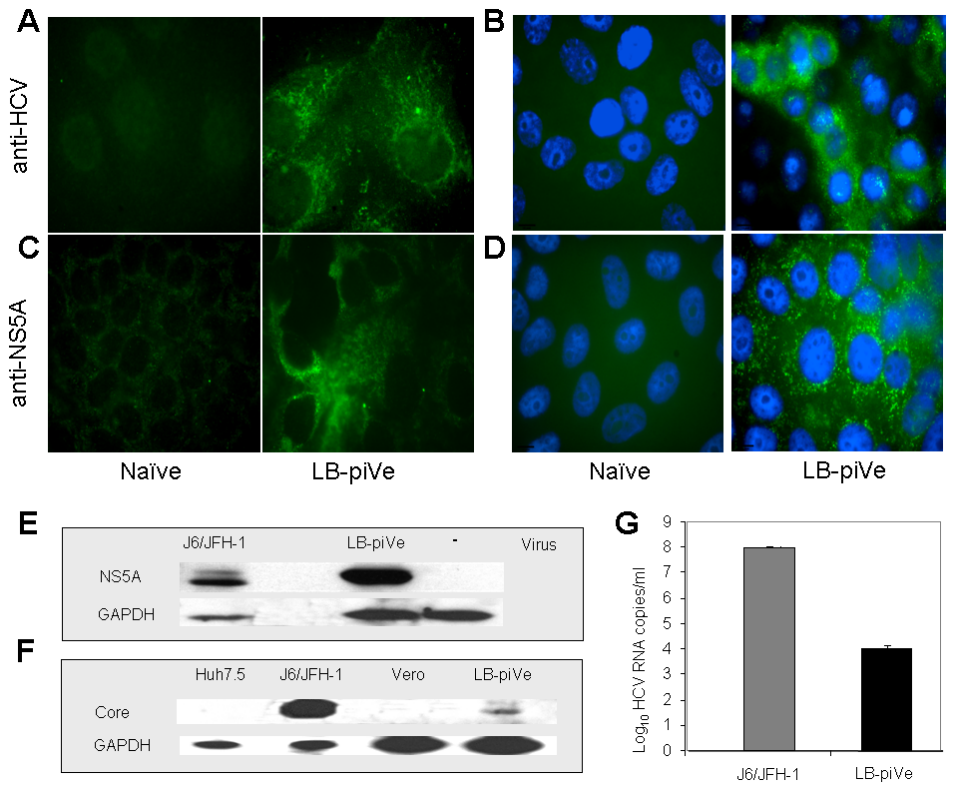
Genotype 1b-persistently infected VeroE6 cells express HCV antigens. Immunostaining of naïve VeroE6 (A–D, *left panels*) and LB-piVe cells (A–D, *right panels*). LB-piVe cells (A, C) and LB-piVe-enriched, panned cells (B, D). (E, F) Immunoblot of LB-piVe and J6/JFH-1-infected cell extracts, stained with anti-NS5A antibody (E) or anti-Core antibody (F). (G) HCV RNA extracted from filter-clarified supernatants from J6/JFH-1-infected Huh7.5 and VA RNA_I_-transfected LB-piVe cells, quantitated by real-time PCR. Error bars, ±SD.

Based on our previous finding that VA RNA_I_ enhanced HCV replication in the replicon system [20], we hypothesized that virus growth in cell culture may also be inhibited by IFN-induced pathways. Our approach was to employ VeroE6 cells, which contain a homozygous-allelic deletion of the IFN-α/β genes [24], [25], yet retain the ability to express IFN-induced genes such us ADAR1 and PKR, which can be activated during virus infection. Cells were transfected with a plasmid encoding VA RNA_I_ (pVA; [26], [27]), and then inoculated once with HCV genotype 1b infectious human plasma, LB [28], [29] (**Figure 1**) or with genotype 1a infectious chimpanzee serum [30] (see **Text S1** and **Table S1**). Normal human serum was used as a negative control. Infected cells were passaged (division ratio of 1∶6) every seven days for 20 weeks with weekly pVA re-transfection (**Table 1**). HCV RNA was detected sporadically in the virus-infected cells after week 20. Nevertheless, passages were continued weekly in the absence of VA RNA_I_. Surprisingly, after 2 years of passage in culture in the absence of VA RNA_I_, HCV RNA was detected consistently, indicating that the virus was able to establish a persistent infection. No virus was detected after 20 weeks in the control experiment that was infected with normal human serum. The possibility that the positive PCR results were due to RNA carry-over is extremely low since the cells had been diluted ∼1.94×10^96^ after 2 years in culture. LB-plasma persistently infected VeroE6 cells (LB-piVe cells) were screened with anti-human- and anti-monkey-specific primers to ensure that the cultures were not contaminated with human cells (data not shown).

**Table 1:** HCV persistent infection in VeroE6 cells.

Week	PCR		Nested PCR	
	LB-piVe	NC	LB-piVe	NC
1	−	−	±	−
2	−	−	±	−
3	−	−	+	−
4	−	−	+	−
5	+	−	+	−
6	ND	−	ND	−
7	−	−	+	−
8	+	−	+	−
9	+	−	+	−
10	+	−	+	−
11	±	−	+	−
12	ND	−	ND	−
13–20	−	−	−	−

VeroE6 cells were transfected with pVA*ls*6 (encoding VA RNA_I_) or pcDNA3, and then infected with genotype 1b-infected human plasma [28], [29], at week 0. For each of 20 weeks the cells were diluted 1∶6, split and re-transfected with pVA*ls*6. Cells were harvested and analyzed weekly for detection of HCV RNA by RT-PCR (PCR, first 40 cycles) followed by nested PCR [62]. Positive, (+); weakly positive, (±); negative (−); negative control pcDNA3, (NC); not determined, (ND). The limit of detection for this assay was evaluated [62] and calculated at 10^3^ RNA copies/ml.

Sequence analysis showed that the persistent virus (LB-piVe virus) shares 99.7% amino acid homology with the parental genotype 1b virus and contains only 10 amino acid changes in the nonstructural region. Sequences have been deposited into GenBank. A representative nucleotide sequence of the LB-piVe virus, aligned with the parental virus sequence and a prototype genotype 1b virus is shown in **Figure S1**. The complete sequence alignment and reverse genetics studies are being conducted and will be presented for publication in the future.

### LB-piVe cells express HCV viral antigens

To visualize HCV antigen expression, we stained LB-piVe fixed cells with polyclonal anti-HCV serum [30] (**Figure 1A and B**) or anti-NS5A monoclonal antibodies (**Figure 1C and D**). To increase the sensitivity of the immunofluorescence assay, we enriched the cell culture by selecting virus-containing, antigen-expressing cells (**Figure 1B and D**) using a cell panning procedure (see Materials and Methods). LB-piVe cells expressed HCV antigens in both perinuclear and cytoplasmic regions of the cells as expected (**Figure 1A–D** **,** ***right panels***). The results suggest that the addition of VA RNA_I_ may broaden cellular tropism by allowing persistent growth and replication of HCV from plasma in non-hepatic VeroE6 cells.

Western blot analysis of LB-piVe (after two rounds of cell panning) and J6/JFH-1-infected cell extracts demonstrates that HCV proteins were expressed at detectable levels (**Figure 1E and F**). Because the proportion of immunofluorescent cells was low, we then compared the levels of viral RNA in filter-clarified supernatants from LB-piVe panned cells versus J6/JFH-1-infected Huh7.5 cells (**Figure 1G**). J6/JFH-1 yielded 9.2×10^7^ RNA copies/ml, while LB-piVe yielded 1×10^4^ RNA copies/ml; (see **Figure 1G**). Persistent infection could only be maintained at a low viral titer, as attempts to obtain the higher viral yields by cell panning (**Figure 1B and D**) resulted in viral instability due to cell cytolysis (data not shown).

### Quantitation of viral titers by a CPE (cytopathic effect)-based assay

**Figure 2:**
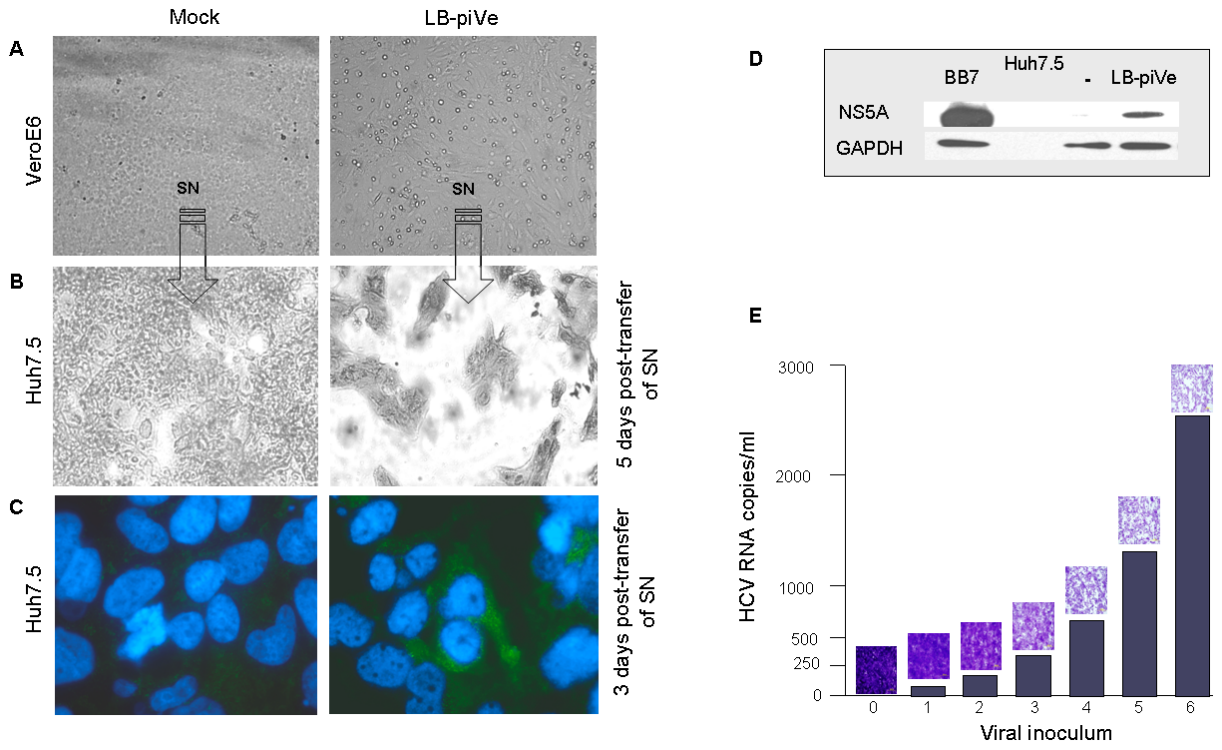
HCV growth can be measured by observing cytopathic effects. Light microscopy of (A) mock-infected VeroE6 (*left panel*) and LB-piVe cells (*right panel*) and (B) crystal violet-stained Huh7.5 cells 5 days after transfer of supernatant from A. (C) Mock-infected (*left panel*) and LB-piVe-infected Huh7.5 cells (from B, *right panel*) immunostained with anti-Core antibodies at 3 dpi. (D) Immunoblot of BB7 replicon containing Huh7.5 cells or LB-piVe-infected Huh7.5 cell extracts, stained with anti-NS5A antibody. (E) Histogram showing quantitative RNA titer (HCV RNA copies/ml, see **Materials and Methods** for details on HCV RNA quantification) and corresponding micrographs of crystal violet stained LB-piVe-infected Huh7.5 cells at 5 dpi (mag 200×).

Interestingly, we observed evidence of CPE in LB-piVe cells after 2 years in culture (**Figure 2A** **, right**). To demonstrate that the virus from the persistently-infected cells was infectious, filter-clarified culture supernatants from LB-piVe cells were used to inoculate naïve Huh7.5 cells. The infected Huh7.5 cells demonstrated enhanced CPE compared to the parental LB-piVe cells, and resulted in gross cell death after 5 days (**Figure 2B** **, right**). Viral antigens were detected at 3 days post-transfer of supernatants by immunostaining the infected Huh7.5 cells (**Figure 2C** **, right**) and also by immunoblotting Huh7.5 cell extracts (**Figure 2D**) with anti-NS5A antibody. The level of CPE observed in Huh7.5 cells (**Figure 2E** **, micrographs**) was directly related to the amount of viral RNA in the inoculum (**Figure 2E** **, histogram**). Taken together, our results show that viral infectivity can be transferred from the persistently infected cell line, LB-piVe, to naïve hepatic cells and that the level of CPE correlates with the level of input viral RNA.

Based on these unique characteristics of LB-piVe, we developed a CPE-based end-point dilution assay for quantification of viral titers. Naïve Huh7.5 cells were plated in 96-well plates and then infected with serial dilutions of virus-containing filter-clarified supernatants (see Materials and Methods). Five days post-infection (dpi), cells were observed by light microscopy and those wells showing CPE were assigned a positive result. The 50% tissue culture infectious dose (TCID_50_) was calculated using the method of Reed and Muench [31].

### Virus neutralization

**Figure 3:**
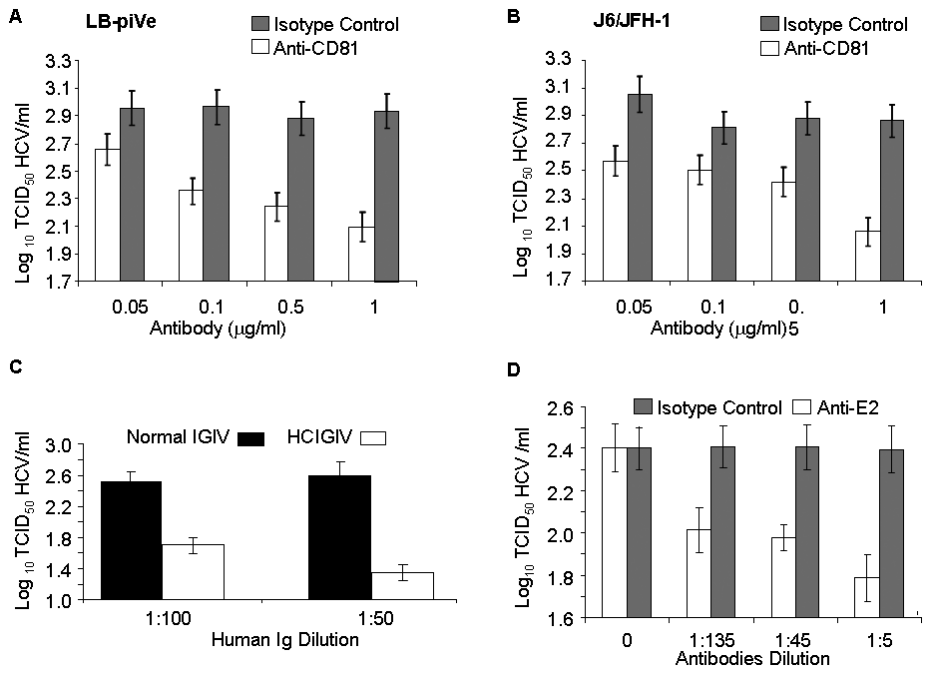
Virus neutralization by anti-CD81 and anti-HCV antibodies. (A) Huh7.5 cells were pre-incubated with anti-CD81 before infection with filter-clarified supernatants from LB-piVe cells or (B) J6/JFH-1-infected cells. M2, isotype-control antibody. J6/JFH-1 was titrated by an end-point dilution assay using indirect immunofluorescence. Wells were scored positive if at least 1 positive cell was detected. (C) LB-piVe was neutralized by incubation with human anti-HCIGIV [35] or (D) anti-E2 monoclonal antibodies [36]. LBpiVe virus titers in A, C and D were determined by a CPE-based TCID_50_ assay. HCV titers were calculated using the method of Reed and Muench [31]. Error bars, ±SD.

To further confirm that the CPE was linked to virus infection, we employed the end-point dilution assay (based on visualization of cell death) to study virus neutralization. Huh7.5 cells were first incubated with antibodies to the putative viral receptor CD81 [32]–[34] and then infected with serial dilutions of filter-clarified supernatants of LB-piVe (**Figure 3A**) or J6/JFH-1 (**Figure 3B**). Viral titers were determined as described in Materials and Methods. This study showed that anti-CD81 antibodies reduced genotype 1b LB-piVe viral titers by ∼1×log_10_ (**Figure 3A**), similar to that observed for the genotype 2a virus J6/JFH-1 (**Figure 3B**). Pre-incubation of LB-piVe virus with HCV-specific immunoglobulin intravenous (HCIGIV) [35] (**Figure 3C**) or anti-E2 monoclonal antibodies [36] (**Figure 3D**) also inhibited virus growth similarly. However, pre-incubation of LB-piVe virus or J6/JFH-1 with normal IGIV or an isotype-matched negative control antibody did not affect viral titers. It may be noted that the anti-E2 monoclonal antibodies were generated to genotype 1a recombinant E2 proteins, including the hypervariable region. Consequently, their ability to neutralize a genotype 1b virus could be limited to some extent as reflected by the 60% decrease in viral titers observed. These neutralization experiments demonstrate that Huh7.5 cell death resulted from the transfer of virus from the LB-piVe cells, and that infection and viral spread in Huh7.5 cells was blocked by the addition of HCV-specific antibodies.

**Figure 4:**
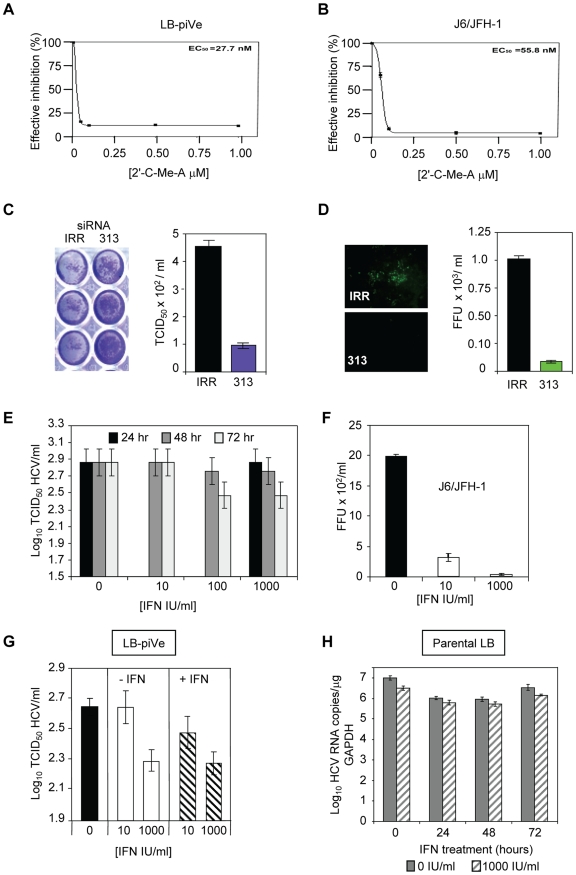
Inhibition of HCV by antivirals. (A) LB-piVe cells (B) and J6/JFH1-infected Huh7.5 cells were treated with increasing concentrations of 2′-C-Me-A. The EC_50_ values were evaluated from dose response curves employing GraphPad Prism 3.0 software. (C) Non-specific siRNA (IRR) [64] or HCV-specific siRNA (313) [38] transfected Huh7.5 cells were inoculated with LB-piVe and stained with crystal violet (*left*). Filter-clarified culture supernatants were titrated by a CPE-based end-point dilution assay (*right*). (D) Non-specific siRNA (IRR) [64] or HCV-specific siRNA (313) [38] transfected Huh7.5 cells were inoculated with J6/JFH-1 and stained with anti-Core antibodies at 3 dpi (*left*). Fluorescent foci were counted in triplicate wells, and titers were calculated as the mean number of foci per ml (FFU/ml, *right*). (E) LB-piVe cells were transfected with pVA and then treated with 0, 10, 100 and 1000 IU/ml of Universal Type 1 IFN for 24, 48 and 72 hr. Viral titers were determined as in B. (F) Huh7.5 cells were inoculated with J6/JFH-1 and treated with 0, 10, and 1000 IU/ml of Universal Type 1 IFN for 72 hr. Viral titers were determined by infecting Huh7.5 cells in the absence of pVA. (G) LB-piVe titers in the absence of pVA, determined in naïve Huh7.5 cells with (+IFN) or without (−IFN) the addition of 0, 10, and 1000 IU/ml of Universal Type I IFN to the culture media. (H) Infection of naïve, non-transfected VeroE6 cells with genotype 1b-infectious human plasma (LB; [28], [29]) and treated with 1000 IU/mL IFN. HCV RNA copies were determined per µg of GAPDH RNA. HCV titers were calculated using the method of Reed and Muench [31]. Error bars, ±SD.

We then explored the utility of the CPE-based assay to screen therapeutics by treating virus-infected cells with HCV inhibitors. We used a well characterized inhibitor of the HCV polymerase, 2′-C-Methyl-D-Adenosine (2′-C-Me-A) [37]. J6/JFH-1 and LB-piVe infected cells were incubated with complete growth medium containing a range of 2′-C-Me-A, from 0.05 to 1 µM. Titers were determined as described in Materials and Methods. The results showed that LB-piVe growth was reduced after treatment with 2′-C-Me-A (**Figure 4A**), with a 50% effective concentration (EC_50_) value in the nanomolar range, comparable to that observed for J6/JFH-1 (**Figure 4B**). Additionally, we tested an HCV-specific small inhibitory RNA to knock-down viral titer (siRNA 313; [38], for details see Materials and Methods). In this assay, siRNA 313 inhibited CPE caused by LB-piVe virus by ∼80% (**Figure 4C**), while J6/JFH-1 was inhibited by >90% (**Figure 4D**).

When LB-piVe cells were treated with IFN, the virus continued to replicate. We measured LB-piVe viral titers in cells that were treated with 0, 10, 100 or 1000 IU/mL of IFN for 24, 48 and 72 hr (**Figure 4E**). LB-piVe titer decreased slightly only at 72 hr with 100 or 1000 IU/mL, but the values were not significantly lower than for 48hr. In contrast, J6/JFH-1 titer decreased by 5-fold when treated with 10 IU/mL for 48 hr and there was no detectable virus with 1000 IU/mL (**Figure 4F**). To ensure that the LB-piVe cells had not become insensitive to IFN treatment, we measured LB-piVe titers in Huh7.5 cells that were also treated with IFN (**Figure 4G**). There was no significant effect on LB-piVe titers by treating the Huh7.5 cells with IFN for 5 days at 10 IU/mL and no change after treatment with 1000 IU/mL. A decrease of 0.2 log_10_ was observed when comparing 10 IU/mL vs 1000 IU/mL over 5 days (**Figure 4G**). This small difference may be attributed to the long incubation period. These results indicate that the LB-piVe virus and not the persistently infected cells are relatively IFN resistant as would be expected for a natural genotype 1b virus isolate. To ensure that the virus had not acquired IFN resistance through passage in culture, we compared the effects of IFN treatment on the parental virus (LB) with the persistent virus in VeroE6 cells (**Figure 4H**). There was little effect after treating the LB virus for 24, 48 or 72 hr, demonstrating that this parental genotype 1b strain was relatively IFN resistant, as expected.

These inhibition studies demonstrated that the LB-piVe virus was sensitive to HCV-specific inhibitors and that the CPE-based assay provides an easy and quantitative method for measuring the efficacy of antiviral compounds. Furthermore, the LB-piVe virus behaved like the wild-type parental virus and maintained its relative IFN resistance.

### Limitations on HCV growth are alleviated by VA RNA_I_

**Figure 5:**
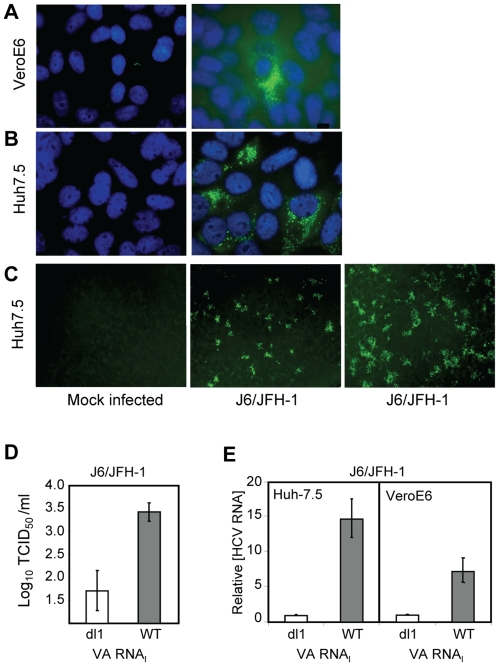
VA RNA_I_ stimulates replication of J6/JFH-1. (A) J6/JFH-1-infected VeroE6 cells and (B) J6/JFH-1-infected Huh 7.5 cells immunostained at 4 dpi with anti-NS5A antibody. Nuclei were visualized using DAPI staining. (C) Huh 7.5 cells (*left*) transfected with a defective (dl1, *center*) [26], [27] or wild-type VA RNA_I_ (+VA RNA_I_, *right*) [26], [27], infected with J6/JFH-1 (*center*, *right*) and immunostained with anti-Core antibodies. (D) Titration of J6/JFH-1 from culture supernatants in C by an end-point dilution assay using indirect immunofluorescence. Wells were scored positive if at least 1 positive cell was detected. The TCID_50_ was calculated using the method of Reed and Muench [31]. (E) J6/JFH-1 RNA (relative to GAPDH RNA) in cell lysates from infected Huh7.5 or VeroE6 cells after transfection with a defective (dl1) or wild-type (WT) VA RNA_I_ [26], [27] and quantitated by real-time PCR. Error bars, ±SD.

VeroE6 cells express the putative receptors for HCV [39]. To elucidate the general growth properties of HCV in these cells, we tested their ability to support replication of the IFN-sensitive genotype 2a virus (**Figure 5**). Cells were mock infected (**Figure 5A and B** **,** ***left***) or J6/JFH-1-infected (**Fig. 5A and B** **,** ***right***), and immunostained at 4 dpi with anti-NS5A antibody. J6/JFH-1 was infectious and replicated in many Huh7.5 cells (**Figure 5B** **,** ***right***) and fewer VeroE6 cells (**Figure 5A** **,** ***right***). An increase in the number, size and intensity of the foci in J6/JFH-1-infected Huh7.5 cells was observed in the presence of wild-type VA RNA_I_ (WT) (**Figure 5C** **,** ***right*** **panel**) but not mutant VA RNA_I_ (dl1) (**Figure 5C** **,** ***center*** **panel**), demonstrating that J6/JFH-1 growth was improved by VA RNA_I_. These data suggest that while the paracrine and autocrine IFN pathways may be defective in VeroE6 cells, additional cellular factors antagonized by VA RNA_I_ are limiting for HCV growth. We also determined the viral titer of the J6/JFH-1-infected Huh7.5 cells, which were transfected with mutant VA RNA_I_ (dl1) or wild-type VA RNA_I_ (WT) (**Figure 5D**). The results showed that wild-type VA RNA_I_ led to an increase in J6/JFH-1 viral titers by >1.5 log units (**Figure 5D** **, WT**), and further confirms that VA RNA_I_ enhances both growth and replication of this genotype 2a virus, probably through inhibition of dsRNA-activated pathways.

The amount of J6/JFH-1 RNA was quantitated (relative to GAPDH) by real-time RT-PCR. Replication of J6/JFH-1 increased by 15-fold (±3-fold SEM) in Huh7.5 cells with VA RNA_I_, whereas VeroE6 cells containing VA RNA_I_ yielded 7-fold (±2-fold SEM) more viral RNA than cells with mutant (dl1) VA RNA_I_ (**Figure 5E**). While the exact mechanism is unknown, a 15-fold increase in viral RNA suggests that the fate of viral RNA in the cells may be affected by the presence of VA RNA_I_, consistent with our previous findings that ADAR1 was inhibited in replicon cells containing VA RNA_I_ [20]. J6/JFH-1 RNA titers were enhanced by VA RNA_I_ twofold in Huh7.5 cells (**Figure 5E** **,** ***left***) over VeroE6 cells (**Figure 5E** **,** ***right***), illustrating preferential growth of J6/JFH-1 in Huh7.5 cells and demonstrating that HCV genotype 2a growth, in addition to genotypes 1a and 1b, is enhanced by VA RNA_I_.

### VA RNA_I_ may increase HCV replication through RNA stability

**Figure 6:**
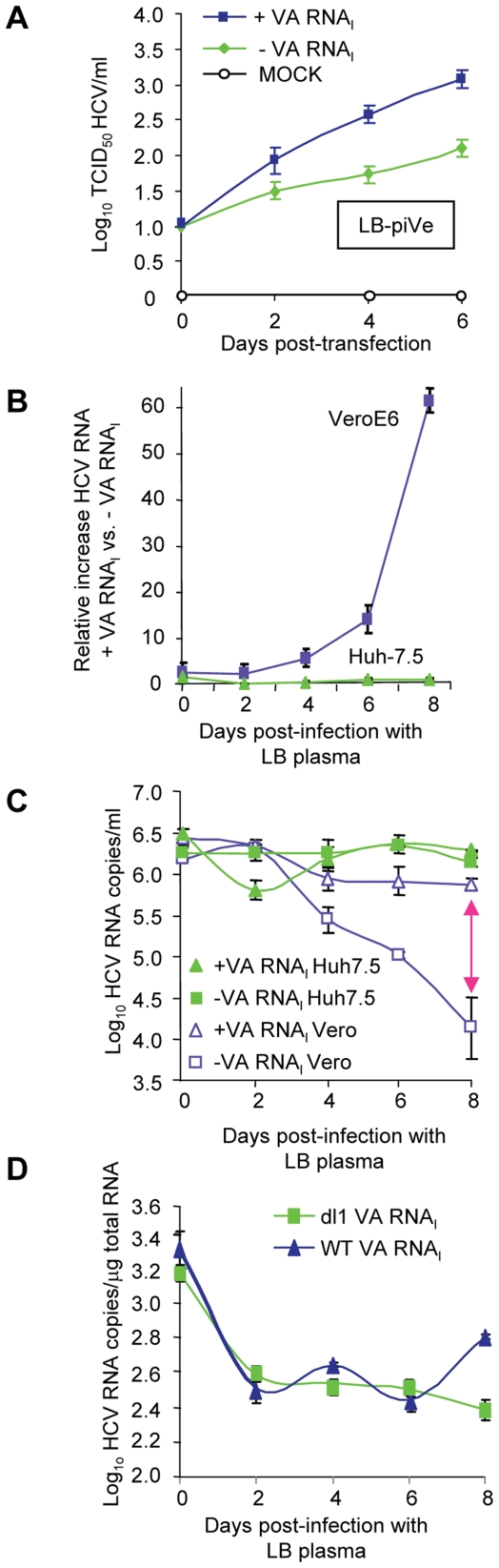
VA RNA_I_ stimulates replication of HCV and increases RNA stability. (A) LB-piVe cells transfected with pVA (+VA RNA_I_ ) or dl1 (−VA RNA_I_). Filter-clarified culture supernatants were collected on days 0, 2, 4 and 6 (post-transfection) for virus titer determination by CPE-based TCID_50_ assay. (B) VeroE6 or Huh7.5 cells transfected with pVA or dl1 and inoculated with genotype 1b-infectious human plasma (LB; [28], [29]). HCV RNA was extracted from cell lysates at the indicated time points and the copy number was determined by quantitative RT-PCR using an HCV standard. Values represent the mean relative increase in HCV RNA relative to GAPDH RNA (+VA RNA_I_ versus −VA RNA_I_). (C) Values in B expressed as log_10_ HCV RNA copies/ml. The double-ended arrow indicates the 60 fold difference in B reflected in the HCV RNA copies. (D) Infection of naïve, transfected VeroE6 cells with genotype 1b-infectious human plasma (LB; [28], [29]) treated with 2′-C-Me-A. HCV RNA copies were determined per µg of total cellular RNA. Error bars, ±SD.

VA RNA_I_ allowed the establishment of a persistently infected cell line and increased growth of LB-piVe (**Figure 6A**) and J6/JFH-1 (**Figure 5A–E**). To evaluate the effects of VA RNA_I_ on the parental virus during the first few days of infection, we examined its effect on HCV RNA stability by comparing the relative increase in viral RNA in VeroE6 cells to that in Huh7.5 cells that were inoculated with the same HCV-positive human plasma (LB, [28], [29]) that was used to establish the persistently infected cell line LB-piVe. Naïve VeroE6 and Huh7.5 cells were transfected with pVA before inoculation with LB plasma (transient infection) (**Figure 6B–D**). RNA was extracted from cell lysates on the days indicated (**Figure 6B, C**) and HCV RNA was measured by quantitative RT-PCR. The relative amount of HCV RNA in pVA-transfected cells versus pVA-untransfected cells (**Figure 6B**) increased 60-fold after 8 days of transient infection in VeroE6 cells. However, in transiently infected Huh7.5 cells, the relative amount of HCV RNA did not increase with the addition of VA RNA_I_ over 8 days (**Figure 6B**), consistent with our inability to obtain a persistently infected Huh7.5 cell line. It may be noted here that the CT values of GAPDH employed as a normalization control in these experiments, were consistent among cells in the presence or absence of pVA. Thus, the dramatic increase in viral RNA in VeroE6 cells may be due to factors other than the variation in transcript levels of GAPDH reported in liver cells [40]–[43]. Furthermore, when the results were expressed in terms of absolute HCV RNA copy number (using an HCV RNA standard curve and measuring copies per ml; **Figure 6C**), the number of RNA copies remained stable in Huh7.5 cells, suggesting that the level of replication may be equal to the degradation of HCV RNA, with or without the addition of VA RNA_I_. In contrast, a precipitous decline in HCV RNA copy number was observed in transiently infected VeroE6 cells in the absence of VA RNA_I_, while the levels remained relatively stable in cells that contained VA RNA_I_ (**Figure 6C**), thus indicating that VA RNA_I_ has an effect on viral RNA over 8 days in VeroE6 cells. We speculate that this effect may be due to; (i) altering the RNA synthesis rate, (ii) altering the degradation rate of HCV RNA molecules other than the input RNA, or (iii) inhibition of an RNA degradation pathway.

Incubation of transiently infected cells with an RNA polymerase inhibitor helped to assess the level of viral RNA in the absence of viral replication. We treated the parental virus with 2′-C-Me-A, which resulted in similar HCV RNA levels when cells were transfected with either WT- or mutant-VA RNA_I_ (**Figure 6D**). Initially there was a decrease in viral RNA (time 0 = input RNA). VA RNA_I_ was not able to stimulate replication in the presence of an inhibitor of HCV polymerase. Additionally, the level of viral RNA did not increase in the presence of wild-type VA RNA_I_, suggesting that viral RNA stability was also not affected by the presence of VA RNA_I_ in the absence of replication. Figure 6 shows that in the presence of VA RNA_I_, viral RNA titer goes down and then levels off; while in the absence of wild-type VA RNA_I_ it continues to decrease (Figure 6C). When the experiment is done in the presence of 2′,C-Me-A, the RNA titer decreases and levels off, independent of VA RNA_I_ (Figure 6D). This is consistent with the mechanism of 2′,C-Me-A, which inhibits new RNA synthesis, however, in this experiment the viral RNA is not degraded 100-fold (*c.f.*, Figure 6C and 6D). We interpret these data as follows: 1) in the absence of viral replication (in 2′,C-Me-A-treated cells), there is less degradation of the viral RNA; 2) in the absence of viral replication (in 2′,C-Me-A-treated cells) there is also the absence of dsRNA (positive strand plus negative strand); and 3) therefore, dsRNA-activated proteins, including ADAR1, would not be activated, leaving VA RNA_I_ with no effect on stability. This is consistent with our ongoing studies that show that only wild-type VA RNA_I_ (that which can bind PKR or ADAR1) is capable of stimulating the replicon (Taylor, unpublished results). Taken together, these results suggest that VA RNA_I_ in the early stages of infection may affect the stability of the viral RNA, either by altering the degradation rate of new HCV RNA molecules or by inhibition of an RNA degradation pathway that may be modulated by viral replication. However, we also cannot exclude the possibility that VA RNA_I_ alters the HCV RNA synthesis rate in the absence of a polymerase inhibitor.

## Discussion

In this study, we have demonstrated that the addition of VA RNA_I_, a known IFN antagonist and inhibitor of dsRNA-mediated antiviral pathways, permitted the persistent growth of a plasma-derived HCV in a cell line that lacks IFN genes. Most of the current knowledge of HCV biology and pathogenesis has been derived from the use of the unique JFH-1 cell culture system, which now allows the study of the complete virus life cycle, including entry, assembly and release. The limitation of this model, however, is that robust viral growth is restricted only to hepatic-derived cell lines such as Huh7.5 and Huh7 cells [44] and only by a genotype 2a replicon-derived virus. The establishment of an alternative model to characterize other HCV genotypes from infected individuals is still needed and is critical for the development of efficient viral therapies to control the disease.

By passaging genotype 1b virus-infected VeroE6 cells for 20 weeks in the presence of VA RNA_I_ and more than 2 years without VA RNA_I_, we generated a persistently infected cell line that expresses HCV antigens at levels high enough to be detected by immunofluorescence and Western blot (**Figure 1A–F** **,** **Table 1**). We found that the LB-piVe virus is highly cytotoxic, and is capable of inducing massive Huh7.5 cell death (**Figure 2B, C and E**); indicating that the virus produced in the persistently infected cells is infectious to hepatocytes. CPE could be blocked by antibodies to CD81 (**Figure 3A**), by anti-HCV-specific immunoglobulins (**Figure 3C**) and by anti-E2 monoclonal antibodies (**Figure 3D**), confirming the link between cell death and viral infection. While neutralization was not as potent using the anti-E2 monoclonal antibodies, we believe that this may be due to the antibodies being raised against recombinant genotype 1a proteins. The genotype differences in the E2 proteins (including hypervariable domains) may be reflected in loss of epitope recognition, thus explaining the 0.6 log_10_ decrease in viral titer.

The LB-piVe virus-mediated CPE has the advantage that it can be assessed visually, and quantified easily and rapidly. This represents a significant improvement over the current genotype 2a HCVcc systems that utilize FFU assays, RT-PCR or reporter assays for quantitation, which are both laborious and time-consuming [45]. In addition, we have demonstrated the utility of this system in virus neutralization studies (**Figure 3A, C and D**) and in testing virus inhibition by well characterized HCV-specific antivirals (**Figure 4A, C, E and G**).

CPE was observed in VeroE6 cells and more-exaggerated CPE was found when filterable supernatants were used to infect Huh7.5 cells. While it was possible to enhance viral titer by panning the LB-piVe cells, and effectively increasing the number of virus-infected cells, the new culture could not survive after several passages. We suspect that the virus cannot be maintained in a culture that demonstrates massive CPE, such as that seen in Huh7.5 cells. This may be the reason that we were unable to obtain persistently infected Huh7.5 cell line, while VeroE6 cells can support persistent HCV infection due to a low-level display of CPE.

HCV- associated cell death has also been reported in Huh7.5.1 cells after infection with JFH-1 when HCV RNA levels reached a maximum [46]. Gene expression profiling of HCV-infected Huh7.5 cells showed both the presence of activated caspase-3 and induction of cell death-related genes, suggesting an association of virus infection with cytopathic effects. Although not yet resolved, it has been postulated that HCV could mediate direct apoptosis by deregulating the cell cycle, which may contribute to liver injury in infected individuals [46]. While still requiring further studies and more comparisons between human pathology and cell culture, we suggest that the LB-piVe system may very well mimic a natural HCV infection in humans and could represent a useful tool to study the intricate process of viral pathogenesis.

VeroE6 cells were also permissive for replication of genotype 2a J6/JFH-1 virus [2] (**Figure 5A–C**). VA RNA_I_ boosted replication and spread in these cells, as shown by the increase in the HCV RNA yield (**Figure 5D, E**). This may be attributable to an increase in viral RNA stability and possibly reflects the type of interplay between host and virus. The presence of VA RNA_I_ allowed for broadened cell tropism by HCV to include non-hepatic cells (**Tables 1** **and S1**), perhaps due to its ability to circumvent the IFN-induced antiviral response. The full-extent of the mechanisms employed by VA RNA_I_ towards overcoming the negative effects of IFN is currently unknown. VA RNA_I_ is important to adenovirus infection and confers virus stability in the presence of IFN and IFN-induced proteins. It has been suggested that VA RNA_I_ has an effect on HCV RNA stability by inhibition of the IFN-induced protein, ADAR1 [20]. When we compared the relative amount of HCV RNA in VA RNA_I_-transfected cells versus -untransfected cells (**Figure 6B, C**) that were infected with HCV-positive human plasma LB, we observed a 60-fold increase in VeroE6 cells. Interestingly, a precipitous decline in HCV RNA was observed in these cells in the absence of VA RNA_I_ (**Figure 6C**). Thus, VA RNA_I_ has an effect in the VeroE6 cells, at least during the first 8 days of infection. We have yet to evaluate possible defects in the RIG-I pathway observed previously in Huh7.5 cells and likely to play a role in early infection [47]. The fact that we did not observe any increase in the relative amount of HCV RNA in Huh7.5 cells after VA RNA_I_ transfection followed by infection with the parental genotype 1b serum-derived virus (**Figure 6B and C**), was unexpected. We suspect that the relatively stable amount of viral RNA reflects extremely low viral replication of the LB virus in Huh7.5 cells. These findings are supported by the evidence that we could not establish a persistently infected cell-line with Huh7.5 cells, suggesting that VeroE6 cells were more permissive for persistent infection, perhaps due to the lack of IFN genes.

VA RNA_I_ was not able to rescue the virus during 2′-C-Me-A treatment and does not stimulate replication nor does it protect the virus from an antiviral that targets the HCV polymerase. We used this RNA polymerase inhibitor to evaluate RNA stability in the absence of viral RNA replication. Since VA RNA_I_ only increased the HCV RNA in the presence of viral replication, we believe that it may perhaps inhibit cellular factors that are activated during viral replication (*e.g.*, dsRNA-binding proteins) and cause instability of the virus. It's possible that VA RNA_I_ interacted with, and therefore blocked ADAR1 and PKR pathways. This would be consistent with our previous findings showing that the HCV replicon was stimulated by knock-down or inhibition of ADAR1 or PKR [20]. Additionally, the inhibition of RNA replication (including loss of negative strand RNA) should inhibit the formation of dsRNA intermediates, thus avoiding the activation of dsRNA-activated proteins that can lead to viral instability [48]–[54]. Again, we cannot exclude the possibility that VA RNA_I_ enhanced viral replication in the absence of the polymerase inhibitor. Taken together, these data suggest that VA RNA_I_ may possibly contribute to establishing a persistent infection in VeroE6 cells; however, the presence of VA RNA_I_ alone is not enough to overcome the cellular antiviral response in Huh7.5 cells. IFN-deficient VeroE6 cells probably provide a more ideal environment for a virus that is, usually, IFN responsive. We suspect that this may be due to the decreased expression of IFN-induced proteins which may actively inhibit HCV replication [20]. Both Huh7.5 cells and VeroE6 cells express PKR and ADAR1, but only the VeroE6 cells lack the IFN genes that induce these proteins. We found that even the persistent virus was stimulated by the presence of VA RNA_I_, suggesting that some of the dsRNA-activated proteins were still expressed and were inhibitory to the virus.

In patients, in general, genotype 2 and 3 viruses are more sensitive to current antiviral therapy than the genotype 1 viruses [55]. Genotype 1b is thought to be the most IFN resistant and the most prevalent in North America, Europe and Japan. However, the HCV replicons (genotype 1b) and J6/JFH1 virus are sensitive to IFN in cell culture. It is not clear why viruses respond to IFN differently *in vivo* versus *in vitro*. Since HCV grows well in VeroE6 cells, especially when assisted by VA RNA_I_, we suggest that endogenous IFNs may limit HCV replication in cell culture.

We suspect that the LB-piVe virus, like the parental LB from which it was derived, was relatively resistant to IFN (**Figure 4E, H**), a property that has not yet been reported in infectious cell culture (**Figure 4G**). While it warrants further investigation, it may be possible that we were able to obtain this virus because VA RNA_I_ was present in the early stages of infection and inhibited the antiviral response generated by viral RNA replication. Our results on the enhancement of virus replication by VA RNA_I_ are clearly consistent with evasion of the antiviral response, and correlate with the observation that susceptibility of human primary hepatocytes to HCV infection could be improved by impairing expression of other IFN signaling factors such us interferon regulatory factor-7 (IRF-7) [**17**]. We suggest that in the early stages of cell culture infection, before viral proteins are in sufficient quantity, the innate immune pathways are active and control infection (RIG-I, PKR, ADAR1, RNaseL, etc.). However, once the virus is given a chance to accumulate, it can overcome these mechanisms of host control, either through the E2, NS5A or NS3 proteins [56]–[58].

Our findings have raised some interesting questions. Future studies with IFN-sensitive viruses, complemented with known IFN-resistant HCV proteins (such as NS5A and E2) using sequences from the LB-piVe virus, are planned. Additionally, the LB-piVe virus will be ideal for evaluating the genes responsible for conferring IFN resistance. We plan to construct an infectious clone and a replicon based on this virus with the aim of evaluating individual genes. At the same time, alignments with the IFN-resistant parental strain of LB with IFN-sensitive genotype 1b replicons may enable the identification of important amino acids that determine IFN resistance. Transient transfection experiments complementing the IFN-sensitive replicons will be among the experiments that will provide insights into the identification of the features that may confer IFN resistance by this genotype 1b virus.

In summary, here we demonstrate that wild-type HCV genotype 1b viruses from human plasma can replicate in African green monkey kidney cells, VeroE6, and that replication of viral genotypes 1a and 2a can be stimulated by the presence of VA RNA_I_. This is a new approach to culturing HCV and the first report of a cell culture system that represents a convenient assay for studying genotype 1b. This is an improvement in terms of utility for research, as the virus can be titrated without employing error-prone, quantitative RT-PCR methods nor arduous immunocytochemistry-based focus forming assays. The availability of the LB-piVe virus raises an exciting possibility; potentially opening a new era of HCV research through the use of a new model system. Moreover, a persistently infected cell line that exhibits CPE provides a novel assay that may be conducive to high throughput development and screening of new antivirals.

## Materials and Methods

### Plasmids

pVA containing the adenovirus 2 virus-associated RNA I (VA RNA_I_) sequence, and VA RNA_I_ mutant dl1 (pVA*dl1*) plasmids, were provided by M. B. Mathews [26], [27].

### Cell culture and transfections

VeroE6 cells (ATCC) were maintained in complete Dulbecco's modified Eagle's medium (DMEM; Invitrogen, Carlsbad, CA) containing 10% heat-inactivated Fetal Bovine Serum (FBS; Hyclone) at 37°C with 5% CO_2_. Huh7.5 cells were provided by C.M. Rice (Rockefeller University, NY) and maintained in complete DMEM containing 10% FBS and non-essential amino acids (Invitrogen). FBS was screened by RT-PCR to ensure the absence of bovine viral diarrhea virus (BVDV). Multiple lots of VeroE6 cells were infected to check for reproducibility. Cells were cultured before transfection in T25 flasks or 6-well plates at a density to provide an overnight confluence of 35%, and transfected with 15–30 µg plasmid vector pVA [26], [27] using DMRIE-C per the manufacturer's specifications (Invitrogen, Carlsbad, CA).

### Hepatitis viruses and titer determination

#### HCV genotype 1b

Cells transfected with pVA were inoculated with 200 µl of plasma containing 10^7^ RNA copies/ml from a genotype 1b-infected patient (LB, [28], [29]) at week 0. Seven dpi, cells were divided 1∶6 and transferred to T25 flasks. The next day, cells were transfected with pVA. The passage and transfection process was repeated, without re-infection, weekly for 20 weeks (weekly results shown in **Table 1**). After 20 weeks, cells were divided 1∶6 weekly without transfection or re-infection for two years. These LB-persistently infected VeroE6 cells were then known as LB-piVe cells. A cytopathic effect (CPE)-based end-point dilution assay was developed for quantification of LB-piVe virus titer. LB-piVe cells were grown for 8 days. Flasks (T75) containing culture medium were frozen (at −80°C) and thawed 3 times. This mixture was cleared by centrifugation and subsequent filtering (0.45 µm), and filter-clarified culture supernatants were obtained. To measure LB-piVe titers, naïve Huh7.5 cells were plated at a density of 5×10^3^ per well in 96-well plates to obtain 60% confluence after 24 hr, and then infected with serial dilutions of filter-clarified supernatants (8 replicates per dilution). Cells were observed by light microscopy at 5 dpi. Wells showing CPE were assigned a positive result. Alternatively, cells were fixed and stained with Crystal Violet (0.1%). The 50% tissue culture infectious dose (TCID_50_) was calculated using the method of Reed and Muench [31].

#### HCV genotype 2a

Genotype 2a virus J6/JFH-1 [2] was provided by C.M. Rice (Rockefeller University, NY) and was titrated by an end-point dilution assay in 96-well plates. Briefly, virus inocula were serially diluted and used to infect 8 replicate wells of naïve Huh7.5 cells growing in microtiter plates. Four dpi, the cells were washed, fixed with cold methanol, probed with a mouse anti-NS5A antibody (Abcam, Cambridge, MA) and a fluorescein isothiocyanate (FITC)-conjugated goat anti-mouse IgG (H+L) (KPL Inc., Gaithersburg, MD), and finally quantitated using an indirect immunofluorescence assay (see below) [59]. Wells were scored positive if at least 1 positive cell was detected. The TCID_50_ was calculated using the method of Reed and Muench [31]. This procedure was followed for the experiments shown in **Figures 3B** **and** **5D**. Alternatively, stained foci were counted in triplicate wells (**Figures 4D and F**), and titers were calculated as the mean number of foci per ml (FFU/ml).

### Preparation of HCV virus stocks

Virus stocks for J6/JFH-1 were prepared by inoculating 1×10^8^ Huh7.5 cells with 1 ml culture supernatant (10^3^ FFU/ml) in serum-free medium [2]. Inoculated cells were grown at 37°C for 12 days. Filter-clarified culture supernatants were obtained as described above. LB-piVe stocks were prepared by growing the persistently infected cells for 8 days at 37°C in T75 flasks and supernatants were collected as for J6/JFH-1.

### Evaluation of infected cultures

#### Indirect immunofluorescence assays

Mock- and HCV-infected cells grown in eight-well Permanox chamber slides (Nunc Inc., Denmark) at 37°C were washed with PBS, fixed with cold acetone for 30 min, and air dried. After incubation with 2% fetal bovine serum in PBS (to block nonspecific binding), cells were probed with mouse anti-NS5A antibodies (Abcam, Cambridge, MA), anti-Core monoclonal antibodies (Affinity Bioreagents, Golden, CO) or Ch1536 serum [30], followed by washing and staining with FITC-conjugated goat anti-mouse IgG (H+L). Slides were mounted with VECTASHIELD mounting medium (Vector laboratories, Burlingame, CA) containing 4′, 6-diamindino-2-phenylindole (DAPI). Fluorescent micrographs were taken with a Zeiss Axiovert microscope at a magnification of 200× (**Figures 2A and B** **; 3G; 4C**) or 1000× (**Figures 1A–G** **; 2C; 4A and B**) with an oil-immersion objective.

#### Western blot analysis

To detect intracellular NS5A, 3×10^6^ infected cells were lysed in 0.2 ml lysis buffer [50 mM Tris, pH 8; 150 mM NaCl; 1% Nonidet P-40; 0.5% Deoxycolate; 0.1% (w/v) sodium dodecyl sulfate (SDS)] containing protease inhibitors (Complete protease inhibitor cocktail; Roche Applied Science, Indianapolis, IN). Cell extracts were clarified by centrifugation, denatured by boiling in Tris-Glycine-SDS sample buffer [60] and resolved on Novex 4–20% Tris-glycine polyacrylamide gels (Invitrogen, Carlsbad, CA) using Tris-Glycine-SDS running buffer [60]. Subsequently, proteins were transferred to Hybond ECL membranes (Amersham, Piscataway, NJ). Membranes were incubated with PBS containing 5% (w/v) nonfat milk and 0.05% Tween-20 (polyoxyethylene sorbitan monolaurate) to reduce nonspecific binding, and then probed with anti-HCV NS5 antibody (Austral Biologicals, San Ramon, CA, at 1∶1000), or anti-glyceraldehyde-3-phosphate dehydrogenase antibody (GAPDH, Trevigen, Gaithersburg, MD, at 1∶3,000). After washing with PBS-tween, membranes were probed with horse radish peroxidase-conjugated secondary antibodies (Kirkegaard & Perry Laboratories, Inc, Gaithersburg, MD), and antigens were detected with SuperSignal West Femto Maximum Sensitivity Substrate (Pierce, Rockford, IL).

#### Cell panning

Polystyrene petri dishes were coated overnight with a 2.5 µg/ml solution of Anti-Human Fc antibodies (KPL Inc., Gaithersburg, MD) in 0,05M Tris-HCl pH = 9.5. Dishes were then washed 3 times with PBS, and incubated 1 hr at room temperature (RT) with a 1∶1000 dilution of an experimental 5% immunoglobulin intravenous (IGIV) preparation made from of anti-HCV positive plasma (HCIGIV) or a 5% IGIV preparation made from anti-HCV negative plasma donations [35]. LB-piVe cells were grown for 5 days in a T150 flask, and then detached by incubation with 0.5 mM EDTA in PBS at 37°C for 30 min. After centrifugation, cells were washed, resuspended in 10 ml of PBS containing 5% FBS and distributed into the panning plates. Following an incubation of 2 hr at RT which allowed antigen-expressing cells to attach, the plates were washed three times gently with PBS/5% FBS and recovered and grown in complete 10% FBS DMEM supplemented with non-essential amino acids (Invitrogen, Carlsbad, CA). This protocol was performed 3 consecutive times.

#### LB-piVe sequence analysis

LB-piVe filter-clarified culture supernatants (300 µl; prepared as described above) were used to extract viral RNA with Trizol LS (as per the manufacturer's directions; Invitrogen, Carlsbad, CA) followed by alcohol precipitation. cDNA was synthesized using primer 9325R (5′-TAGGCACCACATGAACCAG-3′) and AffinittyScript Multiple Temperature Reverse Transcriptase (Stratagene, La Jolla, CA) for one hour at 55°C followed by 15 minutes at 70°C. A first round PCR product was generated with primers (300 nM each) 6038S (5′-CAGCAATACTGCGTCGGCACGT-3′) and D1-1R (5′-TTCTTGGATTTCCGCAGGATCTCC-3′) using TaKaRa LA Taq HS (Clontech Laboratories, Inc., Madison, WI) with the following parameters: 2 minutes at 95°C, 40 cycles with 30 seconds at 95°C, 1 minute at 53°C, 3 minutes at 72°C, and a final extension of 10 minutes at 72°C. For the nested-PCR, 5 µl (1/10) of the first PCR sample was added to a new tube containing 45 µl of TaKaRa LA Taq HS PCR reaction mixture and primers (300 nM each) 6144S (5′-CACTATGTGCCTGAGAGCGACGCC-3′) and D1-2R (5′-TCTCTGACTCCACGCGGGTGATGT-3′). The reaction was carried out using the following parameters: 2 minutes at 95°C, 40 cycles with 30 seconds at 95°C, 1 minute at 56°C, 3 minutes at 72°C, and a final extension of 10 minutes at 72°C. The PCR product was gel purified by using the NucleoSpinR Extract II kit ( Macherey-Nagel, Inc. Easton, PA) and sub-cloned into pCRII-TOPO (Invitrogen, Carlsbad, CA). After transformation of *E. coli* competent bacteria, 10 clones were selected. Both strands of the cloned-PCR product were subjected to direct sequencing by using M13 Forward and Reverse primers. Sequencing reactions were performed with the ABI Prism BigDye Terminator version 3.1 Cycle-Sequencing Kit (Applied Biosystems, Foster City, CA) according to the manufacturer's protocol and analyzed by using the ABI Prism 3100 system (Applied Biosystems, Foster City, CA). Sequences have been deposited into the National Center for Biotechnology Institutes GenBank.

### Real-time PCR assays

#### Determination of HCV RNA titers in filter-clarified culture supernatants

Viral RNA was extracted from 300 µl of LB-piVe filter-clarified culture supernatants (prepared as described above) with Trizol LS (as per the manufacturer's directions; Invitrogen, Carlsbad, CA), followed by alcohol precipitation. RNA was quantitated using a NanoDrop 1000 Spectrophotometer (Thermo Fisher Scientific Inc., Waltham, MA). cDNA was synthesized using primer 200R (5′-CAAGAAAGGACCCGGTCGTC-3′) and AffinittyScript Multiple Temperature Reverse Transcriptase (Stratagene, La Jolla, CA) for one hour at 42°C followed by 15 minutes at 70°C. cDNA samples were tested in triplicate in a 25 µl reaction. Reactions contained 5 µl cDNA, Premix Ex Taq reaction mixture (Clontech Laboratories, Inc., Madison, WI), 300 nM each of primers 124S (5′-CCCTCCCGGGAGAGCCATAG-3′) and 200R, and 200 nM of probe (6FAM- 5′-TCTGCGGAACCGGTGAGTACACC-3′-TAMRA, Applied Biosystems, Foster City, CA). Real-time PCR analysis was performed in an ABI 7300 Sequence Detection System as follows: 1 minute at 95°C, 5 cycles with 20 seconds at 95°C and 1 minute at 60°C, 40 cycles with 20 seconds at 95°C, 30 seconds at 60°C and 31 seconds at 72°C.

RNA standards, run in triplicate, were prepared as described previously [61].

#### Determination of HCV RNA titers in cell extracts

Total cellular RNA was extracted from approximately 10^6^ infected Huh7.5 or VeroE6 cells, using the RNeasy minikit, following the manufacturer's recommendations (Qiagen, Valencia, CA), and quantitated using a NanoDrop 1000 Spectrophotometer (Thermo Fisher Scientific Inc., Waltham, MA). RNA was analyzed by RT-PCR using primers in the HCV 5′ end extending to the Core area [62]. Determination of HCV RNA titers in cells infected with genotype 1b plasma (LB) was performed by real-time RT-PCR analysis as described previously [63]. HCV RNA titers in J6/JFH-1 infected cells were quantitated following identical procedures as for LB, but using a fluorescent probe (FAM-labeled) coding for nucleotides 335–358 designed from published sequences [63]. Relative quantitation of HCV RNA was performed with the Comparative C_T_ Method, using glyceraldehyde-3-phosphate dehydrogenase (GAPDH) as endogenous control, following the manufacturer's protocols and recommendations (Applied Biosystems, Foster City, CA).

### Inhibition of infection by anti-CD81 antibodies

Huh7.5 cells were plated at a density of 5×10^3^ per well in 96-well plates to obtain 60% confluence after 24 hr. Cells were incubated with anti-CD81 (BD Pharmingen, San Diego, CA) or isotype-matched control anti-flag M2 (Sigma, St. Louis, MO) antibodies for 1 hr at 37°C.and subsequently infected with serial dilutions of J6/JFH-1 or LB-piVe filter-clarified supernatants. After 6 hr at 37°C, cells were washed and supplemented with fresh media. Three dpi, J6/JFH-1-infected cells were immunostained with anti-Core antibodies [59]. Wells were scored positive if at least 1 positive cell was detected. LB-piVe-infected cells were observed by light microscopy at 5 dpi. Wells showing CPE were assigned a positive result and titers were calculated as described above.

### Neutralization of LB-piVe by anti-E2 antibodies and HCV-specific immunoglobulins (HCIGIV)

8×10^2^ TCID_50_ LB-piVe were treated for 1hr at 37°C with 5-fold dilutions of a cocktail of anti-E2 monoclonal antibodies [36] or 5µg/ml of isotype-matched control anti-flag M2 (Sigma, St. Louis, MO) antibody. The anti-E2 monoclonal antibodies were produced by hybridomas obtained after immunization of BALB/c mice with E1 and E2 glycoproteins expressed in insect cells [36]. Huh7.5 cells growing in 96-well plates were inoculated with serial dilutions of the neutralization reaction products and incubated for 5 days. LB-piVe titers were determined by a CPE-based end-point dilution assay. To test LB-piVe virus neutralization by immunoglobulins prepared from human plasma, virus was incubated with HCIGIV [35] or HCV-negative IGIV [35], before titrating on naïve Huh7.5 cells.

### Inhibition of viral replication with 2′-C-Methyl-D-Adenosine

2′-C-Methyl-D-Adenosine (2′-C-Me-A) was obtained from Carbosynth Ltd. (Berkshire, UK) and resuspended at 100 mM [37] in dimethylsulfoxide (DMSO). 5×10^3^ Huh7.5 cells were infected with filter-clarified supernatants containing 100 FFU of J6/JFH-1 for 12 hours, washed, and incubated with complete growth medium containing a range of 0.05 to 1 µM 2′-C-Me-A. Three dpi, J6/JFH-1 infected cells were immunostained with anti-Core antibodies [59]. Fluorescent foci were counted in triplicate wells, and titers were calculated as the mean number of foci per ml (FFU/ml). To measure inhibition of LB-piVe growth, 5×10^5^ LB-piVe cells were grown in T25 flasks. After 3 days, 2′-C-Me-A was added to the growth media and cells were incubated for 3 additional days. Filter-clarified culture supernatants from treated LB-piVe cells were titrated in a CPE-based end-point dilution assay as described above. HCV growth in the absence of 2′-C-Me-A was set at 100%. The percentage reduction in the inhibitor treated cells relative to the untreated control was plotted against 2′-C-Me-A concentrations, employing GraphPad Prism 3.0 software. 50% effective concentration (EC_50_) value values were interpolated from the resulting curves. To measure the level of viral RNA in the presence of 2′-C-Me-A, Vero E6 cells growing in 6-well plates were transfected with either WT- or mutant-VA RNA_I_. Four hours post-transfection, cells were treated with media containing 1 µM 2′-C-Me-A overnight and then infected with the parental LB virus. Total cellular RNA was extracted at 0, 2, 4, 6 and 8 days post-infection. Determination of HCV RNA titers was performed by real-time RT-PCR analysis as described previously [62].

### Inhibition of viral replication by HCV-specific siRNA

A chemically synthesized irrelevant oligo, termed siIRR [64] [5′-AAGGACUUCCAGAAGAACAUCTT-3′] and an HCV-specific oligo, termed si313 [38] [5′-CCCGGGAGGUCUCGUAGACTT-3′ ], were obtained from Dharmacon, Lafayette, CO. Huh7.5 cells (2×10^4^) were transfected with 100 nM of siIRR or si313 using DharmaFECT Transfection reagent 1 (Dharmacon, Lafayette, CO) following the manufacturer's protocol and recommendations. One day after siRNA transfection, cells were infected with 200 FFU of J6/JFH-1 or 200 TCID_50_ LB-piVe. Three dpi, J6/JFH-1 infected cells were washed, fixed and stained using an indirect immunofluorescence assay with anti-Core antibodies [59]. Fluorescent foci were counted in triplicate wells, and titers were calculated as the mean number of foci per ml (FFU/ml). LB-piVe infected cells were detected by observing CPE by light microscopy. Culture supernatants of LB-piVe infected cells were titrated by a CPE-based end-point dilution assay on naïve Huh7.5 cells as described above. Viral titers were calculated using the method of Reed and Muench [31].

### Inhibition of viral replication by IFN

LB-piVe cells were grown in T25 flasks at a density to provide an overnight confluence of 40%, and transfected with pVA. One day post-transfection, cells were treated with 0, 10, 100 and 1000 IU/ml of Universal Type 1 IFN (PBL Interferon Source, Piscataway, NJ) for 24, 48 and 72 hr. Filter-clarified culture supernatants were prepared and titrated by a CPE-based end-point dilution assay on naïve Huh7.5 cells as described above. Viral titers were calculated using the method of Reed and Muench [31]. Alternatively, LB-piVe titers were determined in naïve Huh7.5 in the presence of 0, 10, and 1000 IU/ml of IFN.

### Effect of VA RNA_I_ on J6/JFH-1

To show the effect of VA RNA_I_ on J6/JFH-1, Huh7.5 cells were transfected with plasmid vectors pVA or pVA*dl* [26], [27], and infected the following day with 10^5^ TCID_50_. J6/JFH-1 (filter-clarified culture supernatants from Huh7.5 cells that were transfected with wild-type or mutant VA RNA_I_ plasmids). The infected Huh7.5 cells were titrated by end-point dilution in 96-well plates as described above. Wells were scored positive if at least 1 positive cell was detected. The TCID_50_ was calculated using the method of Reed and Muench [31].

### Accession numbers

Sequences can be accessed from GenBank through the NCBI website: H77 (AF009606); JFH-1 (AB047639); adenovirus 2 (AC000007); LB-piVe (FJ976045, FJ976046 and FJ976047).

## Supporting Information

Figure S1 LB-piVe shares sequence homology but not identity with prototype genotype 1b strain. Nucleotide sequence alignment of LB-piVe derived from clones fragments amplified from persistently infected cells. The 3′-UTR amplicons derived from each clone were sequenced bidirectionally. The HCV genotype 1b sequence (EU155326.2) is shown on the third line. Nucleotides in the sequences identical to those of the reference are shown in green, similar nucleotides (purines or pyrimidines) are shown in cyan, deletions are shown as dashes, and different nucleotides are shown in white. Numbering of the nucleotides is according to the EU155326.2 HCV genotype 1b sequence. LB-piVe: virus derived from LB-piVe cells; LB: serum-derived parental virus.
(3.04 MB DOC)

Table S1 HCV genotype 1a replicates in hepatic and non-hepatic cells transfected with VA RNA_I_.
(0.07 MB DOC)

Text S1 Hepatic and non-hepatic cells are permissive to HCV genotype 1a infection if VA RNA_I_ is present.
(0.03 MB DOC)
