# Supplementary material for: Persistent Growth of a Human Plasma-Derived Hepatitis C Virus Genotype 1b Isolate in Cell Culture
Source: PLoS Pathog. 2010 May 20;6(5):e1000910. doi: 10.1371/journal.ppat.1000910 (PMC2873922; doi:10.1371/journal.ppat.1000910)
Supplement: Figure S1 — LB-piVe shares sequence homology but not identity with prototype genotype 1b strain. Nucleotide sequence alignment of LB-piVe derived from clones fragments amplified from persistently infected cells. The 3′-UTR amplicons derived from each clone were sequenced bidirectionally. The HCV genotype 1b sequence (EU155326.2) is shown on the third line. Nucleotides in the sequences identical to those of the reference are shown in green, similar nucleotides (purines or pyrimidines) are shown in cyan, deletions are shown as dashes, and different nucleotides are shown in white. Numbering of the nucleotides is according to the EU155326.2 HCV genotype 1b sequence. LB-piVe: virus derived from LB-piVe cells; LB: serum-derived parental virus. (3.04 MB DOC) [file ppat.1000910.s001.doc]

**Figure S1. LB-piVe shares sequence homology but not identity with prototype genotype 1b strain.** Nucleotide sequence alignment of LB-piVe derived from clones fragments amplified from persistently infected cells. The 3'-UTR amplicons derived from each clone were sequenced bidirectionally. The HCV genotype 1b sequence (EU155326.2) is shown on the third line. Nucleotides in the sequences identical to those of the reference are shown in green, similar nucleotides (purines or pyrimidines) are shown in cyan, deletions are shown as dashes, and different nucleotides are shown in white. Numbering of the nucleotides is according to the EU155326.2 HCV genotype 1b sequence. LB-piVe: virus derived from LB-piVe cells; LB: serum-derived parental virus.


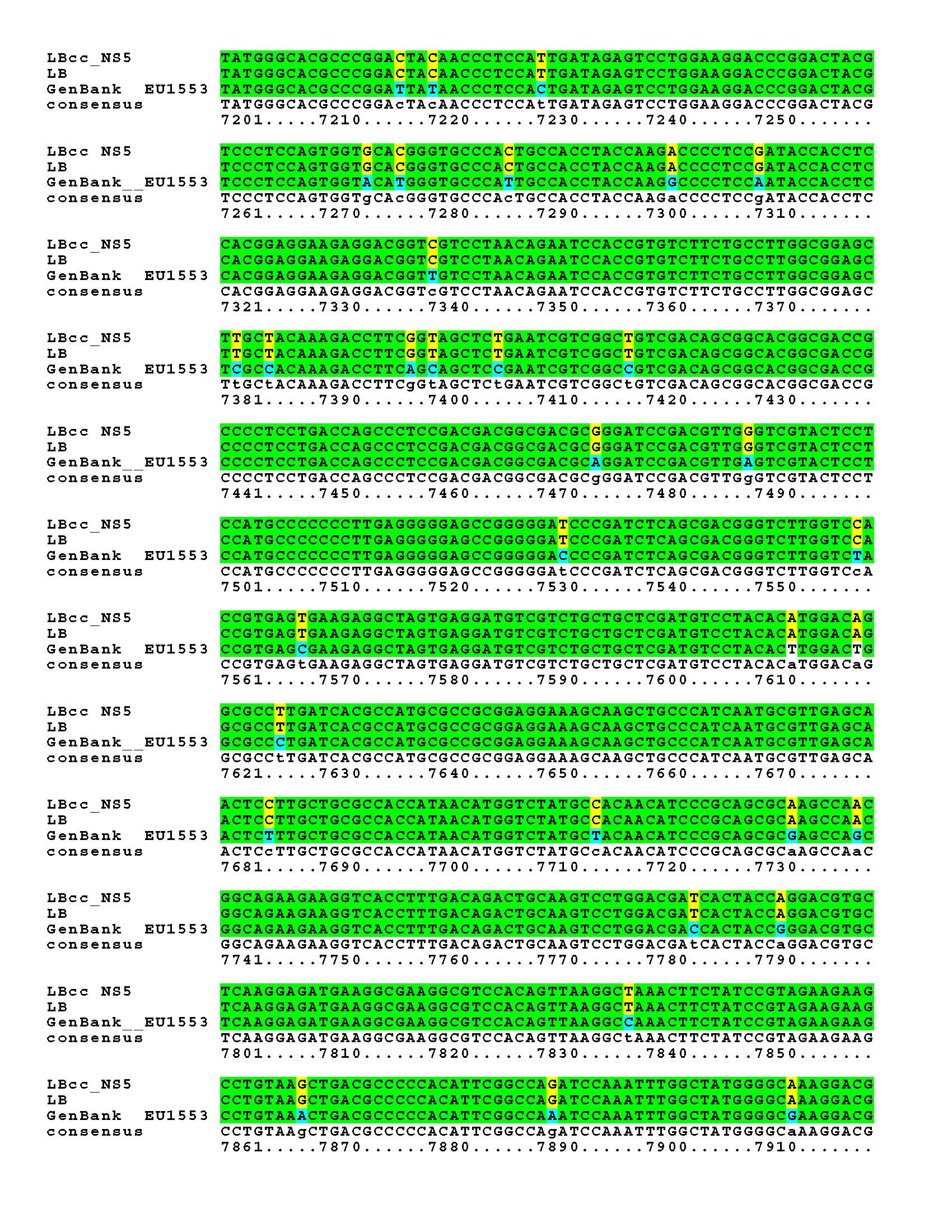


**LB-piVe**

**EU155326.2**

**Consensus**

**LB**

**LB-piVe**

**EU155326.2**

**Consensus**

**LB**

**LB-piVe**

**EU155326.2**

**Consensus**

**LB**

**LB-piVe**

**EU155326.2**

**Consensus**

**LB**

**LB-piVe**

**EU155326.2**

**Consensus**

**LB**

**LB-piVe**

**EU155326.2**

**Consensus**

**LB**

**LB-piVe**

**EU155326.2**

**Consensus**

**LB**

**LB-piVe**

**EU155326.2**

**Consensus**

**LB**

**LB-piVe**

**EU155326.2**

**Consensus**

**LB**

**LB-piVe**

**EU155326.2**

**Consensus**

**LB**

**LB-piVe**

**EU155326.2**

**Consensus**

**LB**

**LB-piVe**

**EU155326.2**

**Consensus**

**LB**
