# Supplementary material for: Persistent Growth of a Human Plasma-Derived Hepatitis C Virus Genotype 1b Isolate in Cell Culture
Source: PLoS Pathog. 2010 May 20;6(5):e1000910. doi: 10.1371/journal.ppat.1000910 (PMC2873922; doi:10.1371/journal.ppat.1000910)
Supplement: Table S1 — HCV genotype 1a replicates in hepatic and non-hepatic cells transfected with VA RNAI. (0.07 MB DOC) [file ppat.1000910.s002.doc]

**Table S1**

**HCV genotype 1a replicates in hepatic and non-hepatic cells transfected with VA RNA_I_.**

| **Cells** | **HCV Infection** | **Transfection** | **PCR** | **Nested PCR** |
| --- | --- | --- | --- | --- |
| **Huh7.5** | No | NC | **-** | **-** |
|  |  | VA RNA_I_ | **-** | **-** |
|  | Yes | NC | **-** | **+** |
|  |  | VA RNA_I_ | **+** | **+** |
| **VeroE6** | No | NC | **-** | **-** |
|  |  | VA RNA_I_ | **-** | **-** |
|  | Yes | NC | **±** | **+** |
|  |  | VA RNA_I_ | **+** | **+** |
| **Vero 76** | No | NC | **-** | **-** |
|  |  | VA RNA_I_ | **-** | **-** |
|  | Yes | NC | **-** | **-** |
|  |  | VA RNA_I_ | **-** | **+** |
| **Vero CCL81** | No | NC | **-** | **-** |
|  |  | VA RNA_I_ | **-** | **-** |
|  | Yes | NC | **-** | **-** |
|  |  | VA RNA_I_ | **-** | **+** |
| **Human lymphocytes** | No | NC | **-** | **-** |
|  |  | VA RNA_I_ | **-** | **-** |
|  | Yes | NC | **-** | **-** |
|  |  | VA RNA_I_ | **-** | **+** |
| **B958** | No | NC | **-** | **-** |
|  |  | VA RNA_I_ | **-** | **-** |
|  | Yes | NC | **-** | **-** |
|  |  | VA RNA_I_ | **-** | **+** |

Cells were transfected with pVA*ls*6 (encoding VA RNA_I_) or pcDNA3, and then inoculated with serum from a chimpanzee infected with a monoclonal HCV genotype 1a (Ch1536 [30]). At 4 days post-infection the cells were lysed and total RNA was analyzed for HCV by RT-PCR and nested PCR [49]. Positive, (+); weakly positive, (±); negative (-); negative control pcDNA3, (NC); not determined (ND). The limit of detection for this assay was evaluated previously [49] and calculated at 10^3^ RNA copies/ml.
