# Supplementary material for: Persistent Growth of a Human Plasma-Derived Hepatitis C Virus Genotype 1b Isolate in Cell Culture
Source: PLoS Pathog. 2010 May 20;6(5):e1000910. doi: 10.1371/journal.ppat.1000910 (PMC2873922; doi:10.1371/journal.ppat.1000910)
Supplement: Text S1 — Hepatic and non-hepatic cells are permissive to HCV genotype 1a infection if VA RNAI is present. (0.03 MB DOC) [file ppat.1000910.s003.doc]

**Text S1**

**Hepatic and non-hepatic cells are permissive to HCV genotype 1a infection if VA RNA_I_ is present.** Several cell types were transfected with a plasmid encoding wild-type VA RNA_I_ (pVA) and then inoculated with 200 μl chimpanzee #1536 serum (3x 10^3^ CID_50_; 10^6^ RNA copies/ml) diluted to 1ml in serum-free media ([30]; **Table S1)**. Chimpanzee #1536 was infected intrahepatically with an infectious consensus clone of HCV genotype 1a. Four days post-infection, we extracted RNA from both culture supernatants and cells, and detected HCV using a single-round of RT-PCR and a highly sensitive nested PCR [49]. We found that Huh7.5 cells were weakly positive (positive in nested PCR only) for HCV RNA. However, when VA RNA_I_ had been transfected into the cells, HCV infection was detected after the first round of 40 cycles of RT-PCR indicating a titer of ≥10^3^ RNA copies/ml [49], and demonstrating improved viral replication in these cells. HCV RNA was also detected in human lymphocytes (PBMCs) and in B958 (Epstein-Barr virus-transformed marmoset B-cells; [52]), but only by nested PCR, when VA RNA_I_ was present as well. This suggests that lymphocytes are permissive to infection and that VA RNA_I_ can increase replication above the limits of detection. Taken together, these data show that hepatic and non-hepatic cells are permissive to HCV genotype 1a infection in the presence of VA RNA_I_.
